# Incidental Acquisition of Foreign Language Vocabulary through Brief Multi-Modal Exposure

**DOI:** 10.1371/journal.pone.0060912

**Published:** 2013-04-08

**Authors:** Marie-Josée Bisson, Walter J. B. van Heuven, Kathy Conklin, Richard J. Tunney

**Affiliations:** 1 School of Psychology, University of Nottingham, Nottingham, United Kingdom; 2 School of English, University of Nottingham, Nottingham, United Kingdom; Emory University, United States of America

## Abstract

First language acquisition requires relatively little effort compared to foreign language acquisition and happens more naturally through informal learning. Informal exposure can also benefit foreign language learning, although evidence for this has been limited to speech perception and production. An important question is whether informal exposure to spoken foreign language also leads to vocabulary learning through the creation of form-meaning links. Here we tested the impact of exposure to foreign language words presented with pictures in an incidental learning phase on subsequent explicit foreign language learning. In the explicit learning phase, we asked adults to learn translation equivalents of foreign language words, some of which had appeared in the incidental learning phase. Results revealed rapid learning of the foreign language words in the incidental learning phase showing that informal exposure to multi-modal foreign language leads to foreign language vocabulary acquisition. The creation of form-meaning links during the incidental learning phase is discussed.

## Introduction

There are many advantages to learning a foreign language (FL), such as a better understanding of another culture or a better chance of employment in an increasingly multilingual society [1]. However, learning a FL can be a difficult and frustrating experience. Informal exposure to a FL requires little effort and benefits FL learners. For example, in childhood, such exposure has been shown to help FL learners acquire a more native like accent as adults [2]. Advanced learners can also improve their FL speech perception by watching a FL film with FL subtitles [3]. Furthermore, exposure to a short FL weather report resulted in an increased sensitivity to the words heard in the weather report compared to other foreign language words [4]. Thus, informal exposure to spoken FL can give rise to speech perception and production benefits. However, can it lead to the acquisition of vocabulary through linking new FL forms with existing meaning representations?

In order to acquire form-meaning links, FL learners are often encouraged to read in the FL [5], [6]. This type of informal exposure provides an incidental learning situation, where a few new words are acquired while learners read for pleasure. However, the incidental acquisition of FL vocabulary through reading is only suitable for more advanced FL learners. In order to be able do derive meaning from context, it is estimated that learners need to know at least 95% of the words in a text [7]. Beginner learners simply do not possess enough FL knowledge to achieve this. A multi-modal situation, which presents both verbal and pictorial information, may be more appropriate for learners of all levels, as in this case, the meaning of the words can be derived from the pictorial information. In such a situation, and with complete beginners it is so far unclear whether form-meaning links can be acquired incidentally.

Here we investigated the effects of a brief multi-modal incidental learning situation on subsequent explicit FL word-learning with complete beginners of the FL. The current study differs from prior studies on FL vocabulary learning (see [4]–[6], [8]–[10] for example) as it focused on incidental learning, with complete novices of the FL, and measured the potential acquisition of FL vocabulary after minimal exposure to the FL. Furthermore, the current study addresses the creation of form-meaning links through a few exposures to new FL word forms with their corresponding pictures.

As studies of incidental FL vocabulary learning have highlighted the need for sensitive measures of vocabulary knowledge [5], [8]–[9], [11], we used a methodology based on the savings paradigm to measure the acquisition of FL vocabulary. The savings paradigm is more sensitive than typical recognition and recall tests [12]–[15] and has been used in recent studies of language attrition to detect traces of knowledge [16]–[19]. The idea of the savings paradigm originally comes from Ebbinghaus who noticed that once something had been learnt, a certain amount of residual knowledge remained in memory (referred to as the “forgetting curve”); this residual memory trace facilitated relearning by reducing the number of trials to criterion, a phenomena now known as “savings” [20]. Importantly, in contrast to prior studies, the present study used the savings paradigm to detect traces of new FL vocabulary knowledge that has not necessarily reached the threshold for explicit recognition or recall.

**Figure 1:**
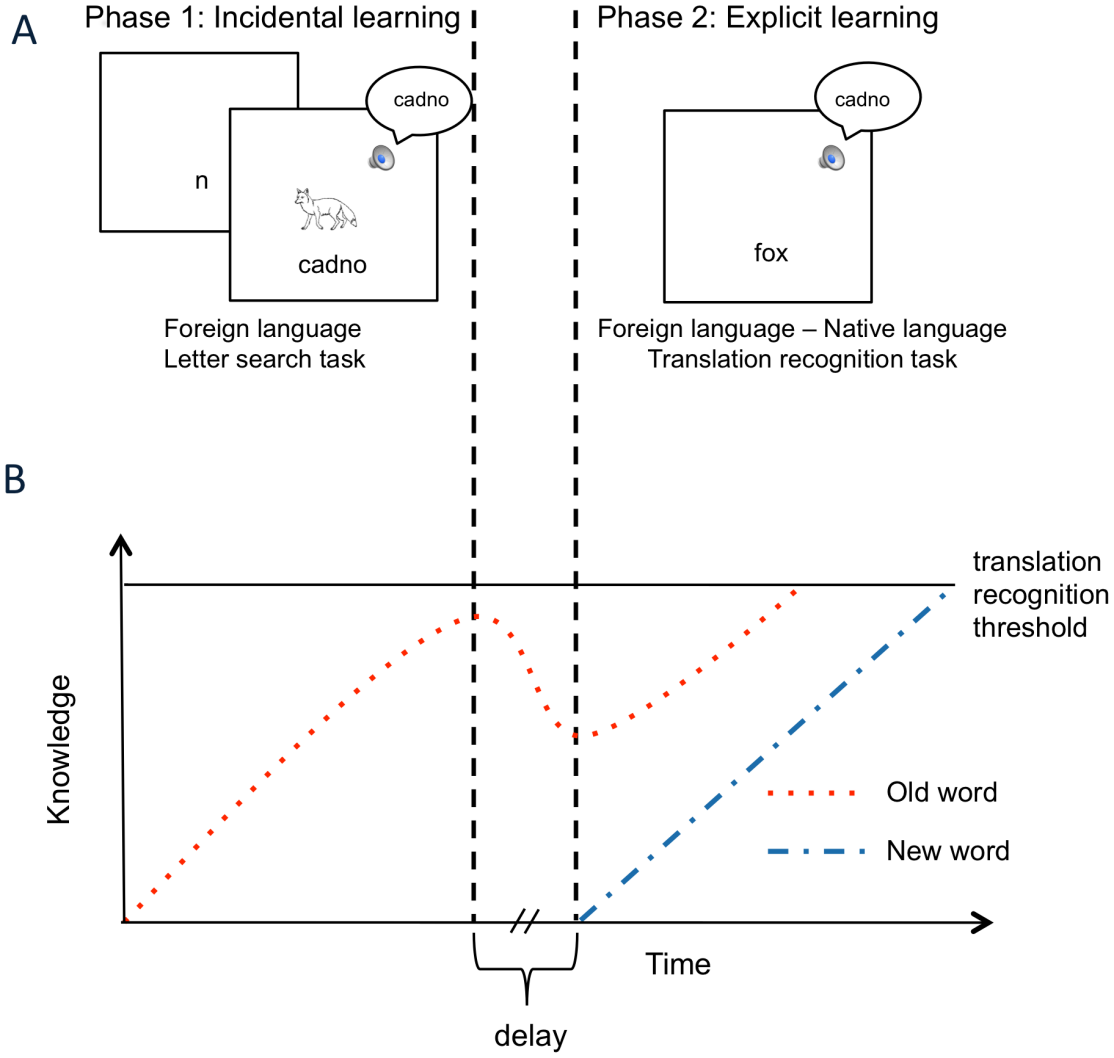
Experimental paradigm (panel A) and predicted effect on foreign language (FL) word knowledge (panel B). In phase 1 (incidental learning), participants were exposed to 40 FL words in a letter-search task in which both the auditory and written forms of a FL (Welsh) word were presented simultaneously with a picture illustrating the meaning of the word (8 repetitions each). In Phase 2 (explicit learning), participants were presented with an auditory Welsh word and were asked to indicate with a button press whether the written English word presented simultaneously on the screen was its correct translation or not. The 40 words from phase 1 (old words) as well as 40 new words were used for this part of the experiment. It was expected that in the incidental learning phase, participants would start building some knowledge about the old words, and that this would help them reach the translation recognition threshold faster for these words then for completely new words during the subsequent explicit learning phase.

As illustrated in Figure 1A, phase 1 of the experiment, the incidental learning phase, made use of multi-modal FL stimuli by presenting auditory and written FL words with a picture illustrating the meaning. Participants engaged in a letter-search task in order to provide an incidental learning situation. Importantly, participants did not know the FL and were unaware that their acquisition of FL vocabulary would be assessed later on. In order to complete the task, participants only needed to attend to the written word form: the auditory word form and the picture were irrelevant for the task. However, the meaning of the FL word could be inferred from the picture. In phase 2, the explicit learning task, participants were asked to learn the meaning of FL words through a translation recognition task. Auditory FL word forms from phase 1 (old words) as well as new auditory FL words not previously encountered were presented simultaneously with an English word that was either the correct or incorrect translation. It was expected that in the incidental learning phase, participants would start building some knowledge about the old words, and that this would help them reach the translation recognition threshold faster for these words then for completely new words during the subsequent explicit learning phase (Figure 1B).

To ensure that differences in performance for the old and the new words in the explicit learning task could be attributed to acquisition rather than to attentional arousal, in the incidental learning phase, a different group of participants (mismatched group) saw picture stimuli that did not match the correct meaning of the words. If attentional arousal leads to an advantage for the old words, the results for this group should not differ from the group where the pictures matched the meaning of the words, as both groups were exposed to the same FL word forms.

Another group of participants (multi-session group) took part in phase 2 of the experiment the next day rather than immediately after phase 1 and they completed the translation recognition task once again one week later. This multi-session group was used to explore whether the incidentally acquired form-meaning links were transitory or became embedded in memory after a relatively long retention interval.

## Methods

### 2.1. Ethics Statement

This research was approved by the School of Psychology Ethics Committee at the University of Nottingham, and all participants gave written informed consent prior to taking part.

### 2.2. Participants

Sixty-six participants took part in the experiment and received payment for their participation. Participants were all native English speakers with no prior knowledge of Welsh. They were split into four groups of participants. Two groups of 16 participants completed phase 1 and 2 of the study in a single-session: matched picture group (mean age 21.6, 11 females) and mismatched picture group (mean age 21.0, 15 females). A multi-session group of 18 participants (mean age 18.9, 15 females) completed phase 1 on the first day of the study, phase 2 the next day, and returned one week later to complete phase 2 once more (one participant from this group did not return one week later and was therefore removed from the analyses). A further 16 participants (mean age 25.0, 12 females) were included as a control group and only completed phase 2 of the study.

### 2.3. Stimuli

Welsh was chosen as the FL because it uses the same script as English but is sufficiently different from English so that participants could not simply guess the meaning of the words based on phonological or orthographic similarity. The stimuli consisted of 80 Welsh words (both the written and auditory forms) and 80 pictures corresponding to these words [21]. The words were split into two sets, and these were matched for category [21], word frequency in English (based on CELEX and British National Corpus) and word length in Welsh. None of the words were Welsh-English cognates. In phase 1 of the experiment (incidental learning phase), participants were exposed to one set of words (counterbalanced across participants and groups) with their corresponding pictures, whilst in phase 2 of the experiment (explicit learning phases), all words were used. For the mismatched picture group, the words were presented with a randomly assigned picture in the incidental learning phase (e.g. a picture of a dog presented with the auditory and written Welsh word “bwrrd” meaning “table”) and presented with the same picture for all the trials in the incidental learning phase. The words used in phase 1 were labeled “old words” and the words participants were exposed to for the first time in phase 2 were labeled “new words”. For the control group, one set of words was also classified as “old words” and the other as “new words” (counterbalanced across participants) to perform the analysis despite all of the words being presented for the first time for this group in phase 2 of the experiment.

### 2.4. Procedure

In phase 1 (incidental learning), participants were asked to perform a letter-search task. In each trial, they were presented first with a letter and then a written Welsh word. Their task was to indicate with a button press whether or not the word contained the letter. Each word was presented 4 times with a letter that was included in it and 4 times with a letter that was not (320 trials in total). Although irrelevant to the task, the corresponding auditory Welsh words and pictures were presented simultaneously with each written Welsh word. Participants were told that the words would be in a FL, but they were not informed that the FL was Welsh.

In phase 2 (explicit learning), participants were presented with each auditory Welsh word and were asked to indicate with a button press whether the written English word presented simultaneously on the screen was the correct translation or not. Each Welsh word was presented once with the correct translation and once with a foil in each block. The foils were chosen randomly from amongst the correct English translations and were different for each block. After each trial, participants received feedback on the screen (“correct” or “incorrect”) and they were instructed to use this feedback to learn the correct translations. At the end of each block (160 trials), the percentage of correct answers was calculated and displayed on the screen, and the task continued until a criterion of 80% correct answers in one block was met or after a maximum of 4 blocks (this was reduced to a maximum of 3 blocks for the multi-session group). For this part of the experiment, participants were informed that they would be asked to learn some Welsh words, however they were not told that some of the words had already been presented in phase 1.

## Results

The number of hits and false alarms in blocks 1 and 2 of phase 2 of the experiment were used to calculate *d'* (d-prime) scores (see [22]) for all groups of participants for both old and new words. As participants had reached criterion in block 2 and therefore did not proceed to block 3, we did not analyze the results of block 3. Furthermore the analyses of block 2 yielded the same results as the analyses of block 1, and therefore we only report the results of block 1 throughout the Results section.

### 3.1. Single-session Groups

#### 3.1.1. Matched vs. mismatched picture groups

*Accuracy*. The overall error rate in the letter-search task of incidental learning phase (phase 1) was low (5.8%).

**Figure 2:**
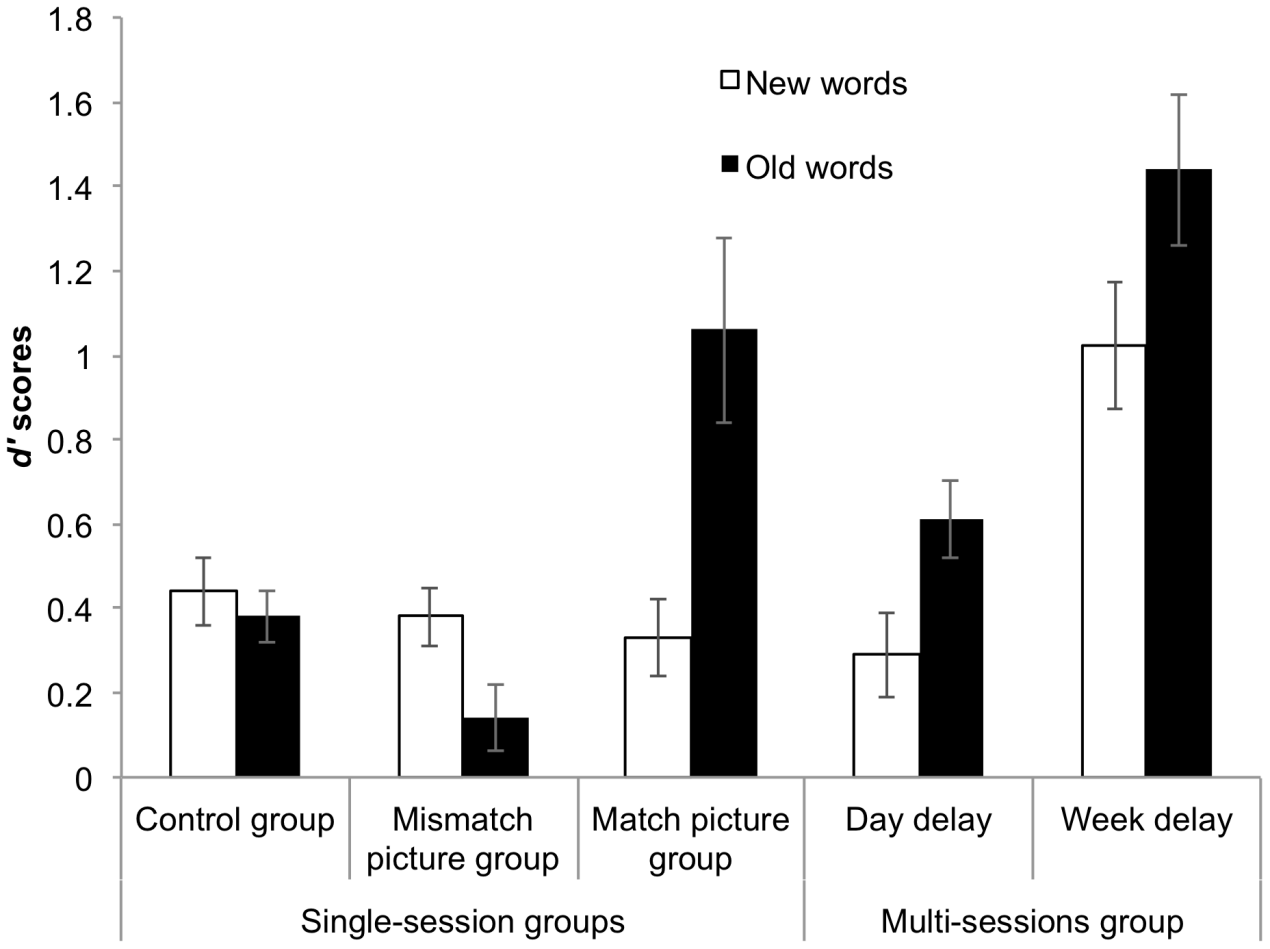
The *d* *'* scores in the translation recognition task. The *d'* scores for old and new words in the translation recognition task (explicit learning phase) in the first block of trials for each group with error bars.

The *d'* scores for block 1 of phase 2 were analyzed using a mixed-design ANOVA with group as a between-subject factor (matched and mismatched picture groups) and word type (new and old words) as a within-subject factor. The results showed significant main effects of word type, *F*(1, 30) = 5.43, *p*<.05, η_p_^2^ = .15, and group, *F*(1, 30) = 8.50, *p*<.01, η_p_^2^ = .22, as well as a significant interaction between word type and group, *F*(1, 30) = 16.87, *p*<.001, η_p_^2^ = .36. This interaction occurred because *d'* scores were significantly higher for old words (*M* = 1.06, *SE* = 0.22) than for new words (*M = *0.33, *SE* = 0.09) in the matched picture group, *F*(1, 30) = 20.72, *p*<.001, η_p_^2^ = .41, whereas for the mismatched picture group *d'* scores were not significantly different between old words (*M* = 0.17, *SE = *0.08) and new words (*M* = 0.38, *SE* = 0.07 ), *F*(1, 30) = 1.58, *p* = .22, η_p_^2^ = .03. Furthermore, independent sample *t-*tests revealed that *d'* scores for the old words were significantly higher in the matched picture group compared to the mismatched picture group, *t*(19.35) = 3.81, *p*<.01, *d* = 1.35. For the new words, there was no difference between the two groups, *t* <1, however, *d'* scores for both groups were significantly higher than chance, *t*(15) = 3.71, *p*<.01, *d* = 0.93, *t*(15) = 5.09, *p*<.001, *d* = 1.27, for the matched and mismatched picture groups respectively. Finally, *d'* scores for the old words in the mismatched picture group were marginally significantly different from chance, *t*(15) = 2.09, *p* = .054, *d* = 0.52 (Figure 2).

**Figure 3:**
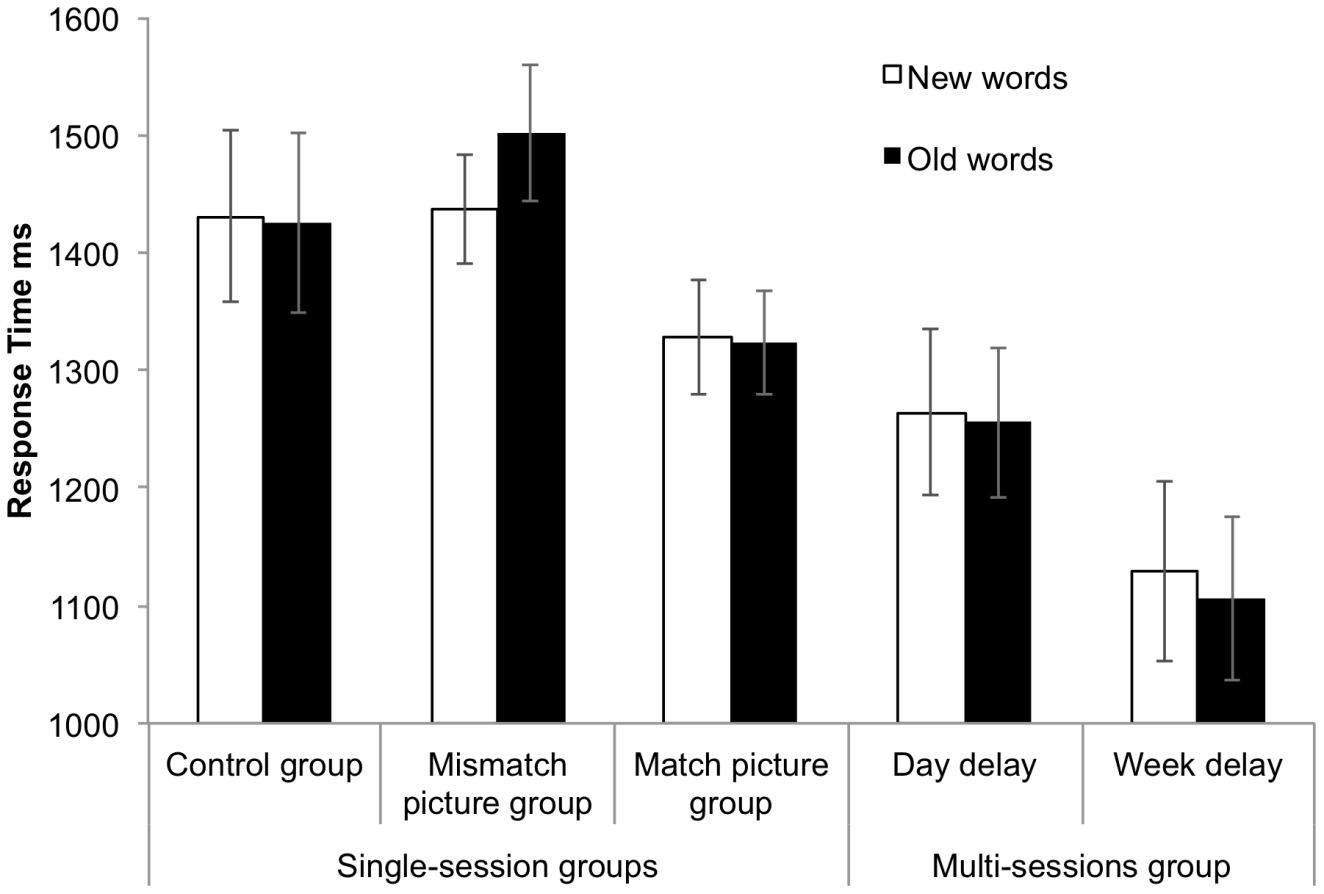
Response times (ms) in the translation recognition task. Response times (ms) for old and new words in the translation recognition task (explicit learning phase) in the first block of trials for each group with error bars.

*Response Times*. A mixed-design ANOVA with group as a between-subject factor (matched and mismatched picture groups) and word type (new and old words) as a within-subject factor revealed a main effect of group, *F_1_*(1, 30) = 4.48, *p*<.05, η_p_^2^ = .13, *F_2_*(1, 79) = 122.59, *p*<.001, η_p_^2^ = .61, but no main effect of word type, *F_1_*(1, 30) = 2.58, *p* = .12, η_p_^2^ = .08, *F_2_*(1, 79) = 2.85, *p* = .10, η_p_^2^ = .04. However, there was a trend towards an interaction between word type and group, *F_1_*(1, 30) = 3.66, *p* = .07, η_p_^2^ = .11, *F_2_*(1, 79) = 6.60, *p*<.05, η_p_^2^ = .08, because the mismatched picture group were significantly slower at responding to the old words (*M = *1502 ms, *SE* = 58 ms) than the new words (*M = *1437 ms, *SE* = 47 ms), *F_1_*(1, 30) = 6.19, *p*<.05, η_p_^2^ = .17, *F_2_*(1, 79) = 9.29, *p*<.01, η_p_^2^ = .11, whereas in the matched picture group there was no significant difference between the responses to old words (*M = *1323 ms, *SE* = 44 ms) and new words overall (*M = *1328 ms, *SE* = 49 ms), *F*s <1 (Figure 3).

An analysis of hits only (correct match trials) revealed that the matched picture group were significantly faster at responding to the old words compared to their responses to the new words, *F_1_*(1, 30) = 6.69, *p*<.05, η_p_^2^ = .18, *F_2_*(1, 79) = 6.45, *p*<.05, η_p_^2^ = .08, whereas there is a trend for the mismatched picture group to be slower at responding to the old words compared to the new words, *F_1_*(1, 30) = 3.23, *p* = .08, η_p_^2.^ = .09, *F_2_*(1, 79) = 3.46, *p* = .07, η_p_^2^ = .04. We do not report the full analyses of response times for hits as it yields the same results as the accuracy analyses.

#### 3.1.2. Control vs. matched picture groups

*Accuracy.* A mixed-design ANOVA with group as a between subject factor (matched picture group and control group) and word type as a within-subject factor (old and new words) revealed a significant main effect of word type, *F*(1, 30) = 9.53, *p*<.01, η_p_^2^ = .24, however, the main effect of group was only marginally significant, *F*(1, 30) = 3.92, *p* = .06, η_p_^2^ = .12. Crucially, the interaction between word type and group was significant, *F*(1, 30) = 13.43, *p*<.01, η_p_^2^ = .31, indicating that the matched picture group performed better on the old words than on the new words, *F*(1, 30) = 22.79, *p*<.001, η_p_^2^ = .42, however this was not the case in the control group (*M* = 0.38, *SE* = 0.06 vs. *M* = 0.44, *SE = *0.08), *F* <1. Furthermore, the matched picture group performed significantly better on the old words than the control group, *t*(17.63) = 3.02, *p*<.01, *d = *1.07, however, there was no significant difference between the two groups for the new words, *t* <1. The *d'* scores in the control group for both old and new words were significantly above chance, *t*(15) = 5.86, *p*<.001, *d* = 1.46, *t*(15) = 5.66, *p*<.001, *d* = 1.42 respectively.

*Response Times.* A mixed-design ANOVA with group as a between-subject factor (matched picture group and control group) and word type (new and old words) as a within-subject factor revealed no significant main effects of group, *F_1_*(1, 30) = 1.41, *p = *.24, η_p_^2^ = .05, *F_2_*(1, 79) = 57.05, *p*<.001, η_p_^2^ = .42, or word type, *Fs* <1, and no interaction *Fs* <1.

#### 3.1.3. Control vs. mismatched picture groups

*Accuracy.* A mixed-design ANOVA with group as a between subject factor (mismatched picture group and control group) and word type as a within-subject factor (old and new words) revealed neither a main effect of word type, *F*(1, 30) = 3.67, *p* = .07, η_p_^2^ = .11, nor of group, *F*(1, 30) = 2.73, *p* = .11, η_p_^2^ = .08 and no interaction between word type and group, *F*(1, 30) = 1.02, *p* = .32, η_p_^2^ = .03.

*Response Times.* A mixed-design ANOVA with group as a between-subject factor (mismatched picture group and control group) and word type (new and old words) as a within-subject factor revealed neither a main effect of word type, *F_1_*(1, 30) = 4.14, *p* = .05, η_p_^2^ = .12, *F_2_*(1, 79) = 2.61, *p* = .11, η_p_^2^ = .03, nor a main effect of group, *F_1_*<1, *F_2_*(1, 79) = 14.16, *p*<.001, η_p_^2^ = .15. However, there was a strong trend for an interaction between group and word type, *F_1_*(1, 30) = 5.84, *p*<.05, η_p_^2^ = .16, *F_2_*(1, 79) = 3.48, *p* = .07, η_p_^2^ = .04, reflecting the slower responses to the old words in the mismatched picture group whereas response times were not significantly different between the old and new words (*M* = 1425 ms, *SE* = 77 ms vs. *M_ = _*1431 ms, *SE = *73 ms) in the control group, *F*s <1.

### 3.2. Multi-session Group

*Accuracy*. Error rates in the letter-search task of phase 1 were again low (6.4%).

The *d'* scores for block 1 of phase 2 were submitted to a repeated-measures ANOVA with word type (new and old) and delay between phases (one day and one week) as within-subject factors. After a one week delay, many participants only completed one block of trials as they reached criterion in block 1, and therefore we did not analyze the results of block 2 for the multi-session group. The results showed a main effect of word type, indicating that *d'* scores were significantly higher overall for old words than for new words (*M* = 1.03, *SE* = 0.12 vs. *M* = 0.65, *SE* = 0.11), *F*(1, 16) = 10.78, *p*<.01, η_p_^2^ = .40. Furthermore, there was a main effect of delay between phases, indicating that *d'* scores were overall higher one week later than the next day (*M* = 1.23, *SE* = 0.15 vs. *M* = 0.45, *SE* = 0.08), *F*(1, 16) = 42.81, *p*<.001, η_p_^2^ = .73. This was expected however, as participants returned to complete the translation recognition task one week later, having already completed 2 or 3 (depending on when they reached the 80% criterion level) blocks of learning on this task the day after phase 1. This explains the overall higher accuracy scores one week later relative to the first block after a day delay. Importantly, there was no interaction between word type and delay between phases, *F* <1, which indicates that participants scored significantly higher for the old words both the next day, *F*(1, 16) = 8.82, *p*<.01, η_p_^2^ = .36 and one week later, *F*(1, 16) = 7.93, *p*<.05, η_p_^2^ = .33. Finally, similarly to the single-session groups, *d'* scores in phase 2 for the new words were significantly above chance, *t*(16) = 2.95, *p*<.01, *d* = 0.72.

*Response Times*. A repeated-measures ANOVA with type of word (old and new) and test time (next day and next week) showed that responses were faster one week later (*M* = 1118 ms, *SE* = 73 ms) than the next day, (*M* = 1260 ms, *SE* = 67 ms), *F_1_*(1, 16) = 6.94, *p*<.05, η_p_^2^ = .30, *F_2_*(1, 79) = 196.98, *p*<.001, η_p_^2^ = .71. However, there was no main effect of word type *F_1_*(1, 16) = 2.43, *p* = .14, η_p_^2^ = .13, *F_2_*<1, and no interaction between word type and test time, *F*s <1.

An analysis of hits only (correct match trials) revealed that participants were significantly faster at responding to the old words compared to their responses to the new words after a one week delay, *F_1_*(1, 16) = 24.53, *p*<.001, η_p_^2^ = .61, *F_2_*(1, 79) = 12.03, *p*<.01, η_p_^2^ = .13, however this was neither the case the next day for block 1, *F_1_*(1, 16) = 1.14, *p* = .30, η_p_^2^ = .07, *F_2_*<1, nor block 2, *F_1_*(1, 16) = 2.93, *p* = .11, η_p_^2^ = .16, *F_2_*(1, 79) = 1.12, *p* = .29, η_p_^2^ = .01.

## Discussion

The results revealed incidental acquisition of FL vocabulary through a brief exposure to multi-modal stimuli. Being exposed to the written and auditory word forms of the FL words, as well as a picture illustrating the meaning of the word, resulted in incidental acquisition of FL vocabulary knowledge as shown by the higher scores for these words in the translation recognition task both immediately after the incidental learning task as well as the next day. In addition, the incidental learning effect remained one week later in the subsequent explicit learning task.

Participants in the mismatched picture group did not benefit from being exposed to the old words in the incidental learning phase, in fact, they suffered from being exposed to the wrong pictures as shown by significantly slower responses to the old words than the new words in the explicit learning phase. This disadvantage caused by the mismatched pictures in the incidental learning phase indicate that this group made form-meaning links that were incorrect. Thus, the higher scores for the words included in the incidental learning phase for the groups exposed to the correct pictures is due to the representation of form-meaning links rather than simple arousal.

An important question is what kind of learning best explains the results of both the matched and mismatched picture groups. Crucially, the observed acquisition of vocabulary reflects more than paired-associate learning between the auditory FL word form and the written native language word form, as this pairing was not presented in phase 1. Here, participants were exposed to the written FL word form (necessary to complete the letter-search task), the auditory FL word form and the meaning of the word via the picture. Written English translations were not presented in phase 1. One explanation for the results is that participants linked the FL word forms with the semantic representation of the words activated by the pictures during phase 1. Then, when the auditory FL word forms were presented in phase 2, participants activated the meaning of the FL words (acquired via the pictures in phase 1) and from there, they could accessed the written native language word form and reach a decision as to whether the translation was correct. Equally, translation recognition could have occurred if the written English word form activated its meaning which in turn was linked to the FL word form. Either way, participants relied on form-meaning links acquired during phase 1 to complete the translation recognition task. This interpretation is compatible with Dobel et al. [23] who also argued that form-meaning links were created during their statistical learning paradigm. In their study, participants were exposed to novel phonological word forms (pseudowords in the native language) in combination with pictures, with correct pairings occurring more frequently than incorrect ones. After completing 5 sessions over 5 consecutive days, participants achieved 90% accuracy in a translation test. The authors concluded that their results showed learning beyond mere stimulus-stimulus association, as the native language word forms used in the translation test were never presented during the statistical learning paradigm.

An alternative explanation for the observed incidental learning effect found here is based on the cascading activation model of speech production (see [24]–[27]). This model predicts that even irrelevant pictures will automatically activate their conceptual representation, and that this in turn will cascade down to activate the lexical representations. Applying this model here would suggest that during the incidental learning phase, the presentation of the line drawings automatically activated the semantic representation for the concept and that this in turn activated the native language lexical representation of the word. As a consequence, it is possible that links were created between the latter and the FL lexical representations (phonological and/or orthographic). However, our task did not involve naming, and it is less clear whether pictures that are irrelevant would activate lexical representations in a task that does not require explicit picture naming (but see [28]). Crucially, even though the cascading model predicts the activation of the native language word form during the processing of the line drawing, the concept still needs to be activated first. Therefore, links could have been created between the FL word forms and BOTH the concept AND the native language word form, i.e., both form-meaning and form-form links. It is also important to remember that all this happened extremely rapidly and in parallel while participants were performing the letter-search task, which required attention to be focused on the FL written word form.

Our current data does not allow us to rule out the second explanation for the locus of the incidental FL vocabulary learning. However, what is certain is that representations in the mental lexicon, either semantic and/or lexical, were automatically activated during the incidental learning phase, and that this in conjunction with the processing of the FL word forms was responsible for the learning. Furthermore, the current study does not enable us to describe the neural mechanisms responsible for the creation and consolidation of form-meaning links, nor was it the aim of the experiment. However, these would likely involve working memory structures (for example the episodic buffer) [29]–[30] with a rapid initial familiarization stage followed by a slower consolidation process as proposed by the complementary learning systems model of memory [31].

Regardless of the precise locus of the form-meaning links, the acquisition of FL vocabulary occurred very rapidly in the incidental learning phase as FL words were only presented 8 times. This is much faster FL word learning with complete novices than found in previous studies. For example, McLaughlin, Osterhout and Kim [10] reported the first evidence of vocabulary learning (the learning of word forms) after 14 hours of exposure to French in a classroom setting. However, learners only became sensitive to the semantic properties of the FL words after approximately 60 hours of exposure. Another interesting study using informal exposure to a 7-minute Chinese weather report showed that some participants became sensitive to the spoken word forms included in the report as opposed to new FL word forms [4]. This study used a similar approach to ours, as the learning was incidental, and the words were presented between 2 to 8 times each in the weather report. However, the results revealed sensitivity to the word forms, which is an early stage of FL word learning, but not to the meaning of the words.

Another important aspect of the present data is the persistence of the incidental learning the next day as well as one week later, which highlights the long lasting impact of informal multi-modal FL exposure in vocabulary learning. Thus, this predicts that multi-modal FL exposure through activities such as games or watching FL films with subtitles could facilitate subsequent formal vocabulary learning even days later.

The new methodology to measure vocabulary acquisition in the current study was based on the savings paradigm. The results indicate that this paradigm is sensitive enough to detect differences in lexical knowledge between words presented in an incidental learning phase and completely new words. This more sensitive measure of vocabulary acquisition could be used in future incidental learning studies as an alternative to traditional recognition and recall vocabulary tests because it does not require explicit vocabulary knowledge.

Overall, our results show for the first time incidental vocabulary learning beyond the form level with complete beginners in the FL. Importantly, this learning persisted the next day as well as one week later. Learning and being able to use FL vocabulary fluently takes a long time, and the present findings show that incidental vocabulary acquisition through multi-modal exposure can play an important role in facilitating this process.

## References

[1] Graddol D (2006) English Next. Available: http://www.britishcouncil.org/learning-research-englishnext.htm.

[2] Au TK, Knightly LM, Jun SA, Oh JS (2002) Overhearing a language during childhood. Psychol sci 13: 238–243. 12009044 10.1111/1467-9280.00444

[3] Mitterer H, McQueen JM (2009) Foreign subtitles help but native-language subtitles harm foreign speech perception. PloS one 4: e7785. 19918371 10.1371/journal.pone.0007785 PMC2775720

[4] Veroude K, Norris DG, Shumskaya E, Gullberg M, Indefrey P (2010) Functional connectivity between brain regions involved in learning words of a new language. Brain Lang 113: 21–27. 20116090 10.1016/j.bandl.2009.12.005

[5] Brown R, Waring R (2008) Incidental vocabulary acquisition from reading, reading-while-listening, and listening to stories. Reading in a Foreign Language 20: 136–163.

[6] Horst M (2005) Learning L2 Vocabulary through Extensive Reading: A Measurement Study. Can Mod Lang Rev 61: 355–382.

[7] Nation ISP (2001) Learning vocabulary in another language. Cambridge: University Press. 477 p.

[8] Kweon S, Kim H (2008) Beyond raw frequency: Incidental vocabulary acquisition in extensive reading. Reading in a Foreign Language 20: 191–215.

[9] Pellicer-sánchez A, Schmitt N (2010) Incidental vocabulary acquisition from an authentic novel: Do Things Fall Apart? Reading in a Foreign Language 22: 31–55.

[10] McLaughlin J, Osterhout L, Kim A (2004) Neural correlates of second-language word learning: minimal instruction produces rapid change. Nat Neurosci 7: 703–704. 15195094 10.1038/nn1264

[11] Bisson M-J, Van Heuven WJB, Conklin K, Tunney RJ (2012) Processing of native and foreign language subtitles in films: An eye tracking study. App Psycholinguist Available on CJO doi:10.1017/S0142716412000434.

[12] Groninger LK, Groninger LD (1980) A comparison of recognition and savings as retrieval measures: A reexamination. B Psychonomic Soc 15: 263–266.

[13] Nelson TO (1978) Detecting small amounts of information in memory: Savings for nonrecognized items. J Exp Psychol - Hum 4: 453–468.

[14] Nelson TO (1985) Ebbinghaus’s contribution to the measurement of retention: savings during relearning. J Exp Psychol Learn 11: 472–479. 10.1037//0278-7393.11.3.472 3160812

[15] MacLeod CM (1988) Forgotten but not gone: savings for pictures and words in long-term memory. J Exp Psychol Learn 14: 195–212. 10.1037//0278-7393.14.2.195 2967343

[16] De Bot K, Stoessel S (2000) In Search of Yesterday’s Words: Reactivating a Long-Forgotten Language. Appl Linguist 21: 333–353.

[17] De Bot K, Martens V, Stoessel S (2004) Finding residual lexical knowledge: The “Savings” approach to testing vocabulary. Int J Bilingual 8: 373–382.

[18] Bowers JS, Mattys SL, Gage SH (2009) Preserved implicit knowledge of a forgotten childhood language. Psychol Sci 20: 1064–1069. 19645694 10.1111/j.1467-9280.2009.02407.x

[19] Hansen L, Umeda Y, McKinney M (2002) Savings in the Relearning of Second Language Vocabulary: The Effects of Time and Proficiency. Lang Learn 52: 653–678.

[20] Ebbinghaus H (1964) Memory: A contribution to experimental psychology. (H. A. Ruger & C. E. Bussenius, Trans.). New York: Dover Publications. (Original work published 1885). 123 p.

[21] Snodgrass JG, Vanderwart M (1980) A standardized set of 260 pictures: norms for name agreement, image agreement, familiarity, and visual complexity. J Exp Psychol - Hum 6: 174–215. 10.1037//0278-7393.6.2.174 7373248

[22] MacMillan NA, Creelman CD (1991) Detection Theory: A users guide. Mahwah: Lawrence Erlbaum Associates. 513 p.

[23] Dobel C, Junghöfer M, Breitenstein C, Klauke B, Knecht S, et al (2009) New names for known things: on the association of novel word forms with existing semantic information. J Cog Neurosci 22: 1251–1261. 10.1162/jocn.2009.21297 19583468

[24] Kuipers J-R, La Heij W (2009) The limitations of cascading in the speech production system. Lang Cogn Process 24: 120–135.

[25] Meyer AS, Damian MF (2007) Activation of distractor names in the picture-picture interference paradigm. Mem Cognit 35: 494–503. 10.3758/bf03193289 17691148

[26] Morsella E, Miozzo M (2002) Evidence for a cascade model of lexical access in speech production. J Exp Psychol Learn 28: 555–563. 12018507

[27] Navarrete E, Costa A (2005) Phonological activation of ignored pictures: Further evidence for a cascade model of lexical access. J Mem Lang 53: 359–377.

[28] Bles M, Jansma BM (2008) Phonological processing of ignored distractor pictures, an fMRI investigation. BMC Neurosci 9: 20. 18267005 10.1186/1471-2202-9-20 PMC2259309

[29] Baddeley A (2000) The episodic buffer: a new component of working memory? Trends Cogn Sci 4: 417–423. 11058819 10.1016/s1364-6613(00)01538-2

[30] Baddeley AD (2002) Is working memory still working? Eur Psychol 7: 85–97.

[31] Davis MH, Gaskell GM (2009) A complementary systems account of word learning: Neural and behavioural evidence. Phil Trans R Soc 364: 3773–3800. 10.1098/rstb.2009.0111 PMC2846311 19933145

